# Mutation screening of *CHD5 *in melanoma-prone families linked to 1p36 revealed no deleterious coding or splice site changes

**DOI:** 10.1186/1756-0500-1-86

**Published:** 2008-09-19

**Authors:** David Ng, Xiaohong R Yang, Margaret A Tucker, Alisa M Goldstein

**Affiliations:** 1 Division of Cancer Epidemiology and Genetics, National Cancer Institute, NIH, DHHS, Bethesda, Maryland, USA

## Abstract

**Background:**

A subset of cutaneous malignant melanoma and dysplastic nevi (CMM/DN) families is linked to 1p36. To date, no CMM/DN susceptibility gene has been identified at this locus. Data from mouse studies identified chromodomain helicase DNA binding protein 5 (*CHD5*) as a tumor suppressor affecting cellular proliferation and apoptosis via the *CDKN2A*/p53 pathway. Based on these findings, we felt it was important to screen *CHD5 *as a familial CMM/DN susceptibility gene.

**Methods:**

Eight unrelated CMM/DN families showing prior evidence of linkage to the 1p36 locus were identified for *CHD5 *mutation screening. One CMM/DN affected and one unaffected individual from each family were selected for sequencing of the *CHD5 *coding exons and their respective intron-exon boundaries. *CHD5 *variants that were identified solely among affecteds in the screening panel were further assessed by sequencing additional affected and unaffected members of these families to determine if the variant co-segregated with the CMM/DN phenotype.

**Results:**

Single nucleotide polymorphisms in the *CHD5 *intronic and coding regions were identified among affecteds in the screening panel. None of these variants completely co-segregated with CMM/DN affection status among these eight families.

**Conclusion:**

There is no evidence to support *CHD5 *as a major melanoma susceptibility gene among the eight CMM/DN families screened.

## Background

Familial cutaneous malignant melanoma results from a complex interplay of genetic and environmental components. The etiologic complexity is compounded by the presence of genetic heterogeneity [1]. Two major susceptibility genes *CDKN2A *(MIM 600160), *CDK4 *(MIM 123829) and a modifier gene *MC1R *(MIM 155555) have been identified [1]. Mutations in *CDKN2A *account for 20–40% of CMM kindreds [2], while *CDK4 *mutations have been identified in < 10 CMM families [3-6]. *MC1R *variants have been associated with melanoma risk among individuals of European descent [7,8] and as a modifier of melanoma risk in *CDKN2A *mutation-positive CMM families [9-11]. Two putative chromosome 1p melanoma susceptibility loci (1p22, 1p36) [12-15] have been identified by linkage analysis in a subset of CMM families. In particular, families exhibiting CMM/DN phenotype showed simultaneous linkage to both 1p36 and 9p21 [15]. To date, no candidate genes have been identified at either 1p22 or 1p36.

Deletion of chromosomal band 1p36 is frequently observed in many human cancers including melanoma [16,17] and has been hypothesized to contain a tumor suppressor gene. In 2007, Bagchi *et al*. [18] engineered a mouse with a deletion or duplication of a genomic interval corresponding to human 1p36. Flow cytometry studies of mouse embryonic fibroblasts revealed that mice with a heterozygous deletion exhibited enhanced cellular proliferation whereas mice carrying a duplication showed enhanced senescence. Gene knockdown/rescue experiments identified chromodomain helicase DNA binding domain 5 (Chd5) as the putative dosage-sensitive gene responsible for the observed haploinsufficiency-associated cellular proliferation and duplication-associated apoptosis. Subsequent experiments showed that Chd5 mediated apoptosis involved p53 and that Chd5 haploinsufficiency led to decreased expression of *CDKN2A *(p16) and p19 (human p14^ARF^**-**alternate-spliced exon 1β transcript of *CDKN2A*, aka *ARF*) [18]. These findings support *CHD5 *(MIM 610771) as a putative candidate gene for familial CMM kindreds mapping to 1p36.

To evaluate if *CHD5 *is a major melanoma susceptibility gene in CMM/DN kindreds showing linkage to 1p36, we selected a panel of 16 individuals from eight CMM/DN families for sequencing of all *CHD5 *coding exons and their respective intron-exon boundaries.

## Methods

### Patient selection

Eight familial CMM/DN kindreds with previous evidence of linkage to 1p36 [14] were selected for *CHD5 *sequencing. The 1p36 linkage among the CMM/DN families were based on three genotyped markers D1S47 (RFLP), Z_max _2.82 at θ = 0.1, D1S160, Z_max _3.71 at θ = 0.1 and PND (RFLP located in the NPPA gene), Z_max _2.0 at θ = 0.1 [14]. D1S47 is located adjacent to CHD5, D1S160 and PND are located 3 and 6 Mb (respectively) centromeric to CHD5. Five of these families showed linkage to both 1p36 and 9p21. Among these five families, four have *CDKN2A *mutations and one has an ARF mutation. Among the three remaining families, two have *CDK4 *mutations and one has no identified mutation in *CDKN2A *nor *CDK4 *(Table 1). The families averaged seven CMM patients (minimum, four). The median age at first CMM diagnosis was 31 years; half the CMM patients had multiple melanoma tumors. One affected and one unaffected family member was selected for *CHD5 *mutation screening except for family G in which two affecteds were selected because both the paternal and maternal lineages showed CMM/DN (Table 1). The affecteds were selected based on sharing the putative 1p36 haplotype and the unaffected were chosen who did not share the 1p36 haplotype. All study subjects are of Caucasian descent. This study was approved by the institutional review board of the US National Cancer Institute and adheres to the tenets of the Helsinki Declaration. Written, informed consent was obtained from all participants.

**Table 1:** *CHD5 *mutation screening panel from eight CMM/DN kindreds linked to 1p36

Family	Individual	Affection	CMM+DN	Mutation status
R	3002	Unaffected	No	None
R	3003	Affected	Yes	*CDK4+*
K	1001	Affected	Yes	*CDKN2A+*
K	1003	Unaffected	No	None
D	1001	Affected	Yes	*CDKN2A+*
D	1003	Unaffected	No	None
G	2005	Affected	Yes	*CDKN2A+*
G	2006	Affected	Yes	None
J	1002	Affected	Yes	*CDKN2A+*
J	3005	Unaffected	No	None
AH	3005	Affected	Yes	ARF+
AH	3006	Unaffected	No	None
S	1001	Affected	Yes	*CDK4+*
S	1003	Unaffected	No	None
A2	1001	Affected	Yes	None
A2	1019	Unaffected	No	None

*ARF*, *CDKN2A *alternative-spliced exon 1β transcript, *CDKN2A*, cyclin-dependent kinase inhibitor 2A, *CDK4*, cyclin-dependent kinase 4, CMM, cutaneous malignant melanoma; DN, dysplastic nevi.

### Mutation analysis

Genomic DNA from a panel of 16 individuals (Table 1) was analyzed for *CHD5 *mutations by bi-directional sequencing of all coding exons. Intron-exon boundaries were determined by aligning the reference *CHD5 *mRNA (NM_015557) sequence to the genomic sequence (AL031847, AL035406) with NCBI Spidey [19]. We looked for potentially damaging changes that affect splice donor/acceptor sites or caused in- or out-of-frame deletions/duplications, non-conservative amino acid coding changes and nonsense substitutions. *PCR *primers were designed to amplify exons and flanking introns. Primer sequences and PCR conditions for each exon are detailed in Table 2. Taq Gold (Applied Biosystems, Foster City, CA) or Advantage GC-2 polymerase (Clontech, Mountain View, CA) were used to amplify the genomic DNA from each subject. Sequencing reactions were performed on PCR purified products using BigDye v3.1 chemistry and analyzed with an automated 3130XL Genetic Analyzer (Applied Biosystems).

**Table 2:** Primers and PCR conditions used for mutation screening of the coding exons of *CHD5*.

**Exon**	**Primer (forward/reverse)**	**Annealing**	**Product size (bp)**
2	5'-CTCTCACTTCACTGGGTTTG-3' 5'-GAAACCCTCAAACTCCAAGG-3'	58	393
3	5'-CTCTGATGATGAGTGGAGTG-3' 5'-AACATACAGGCAAGAGGCTCAG-3'	58	394
4	5'-TTTCCTAGGGTGGGTGAGAATG-3' 5'-TTGCTCAGTCGGTCTGACAGAG-3'	58	400
5	5'-CTCTCTAATCAGGAACCTGG-3' 5'-GGCTTCTCCTATAGGGTCTGAAAG-3'	58	441
6	5'-CCCTTTCCTTATTGGGTAACCG-3' 5'-GCCCCAGCTAGTTTGTAATG-3'	58	335
7	5'-GAATCACAGAGAGCACTGTG-3' 5'-TCCTTGTTCTTTCCTTACTGGG-3'	58	491
8	5'-ACATCTACTCTGTGCCTGTCTG-3' 5'-GCATTCTGCCCCCAAATGAG-3'	58	347
9	5'-TGTAGGGGAGGGAGGGAGTC-3' 5'-TTTTGAGGAGGGCAGGCCTTC-3'	67	387
10	5'-CGTGGTACTGTTCTGACTTG-3' 5'-CTCAGTCAGAGGCGCTTCAG-3'	58	435
11	5'-CTGAAGCGCCTCTGACTGAG-3' 5'-TGCGCTGCACCCATTTTACAG-3'	58	459
12 & 13	5'-TGCCCTTCATCAAACCTGTG-3' 5'-CCTGCACATTCAAGTCTGAG-3'	58	472
14	5'-GAATTGCATGTGCAAAGGCCTG-3' 5'-CGCGTTCCCAGTTGATGATG-3'	58	397
15	5'-CCTTTACTCCCTCTACAAGG-3' 5'-ACCCGTGGTCCCTGAACTAG-3'	58	373
16 & 17	5'-TTGGCTCTTTGTCTCCTGGG-3' 5'-CAGGATGGGCTATTGATCCG-3'	58	504
18	5'-TGAGACGATATCCAGGGCAATG-3' 5'-AAAGCATTAGCCGAGACCTCAG-3'	58	431
19	5'-GGTCTCTCTGTAAATGGGTGCTTG-3' 5'-AAACCTACCATGACAGCCACAG-3'	58	396
20	5'-CAGCACTTGTCTGTTCCCTG-3' 5'-ACACAGTCACATGACCCACATG-3'	58	357
21	5'-GGGTGAGGTTGGAAGCTTTG-3' 5'-GTCTTTAGCTGTTTGTGAGGCTG-3'	58	419
22	5'-TTGCTGCAGTTCCTTCTCTCTG-3' 5'-ATGGCAAGAAGGGCATGAAG-3'	58	311
23	5'-TCCATGCTCTTGAGCTCATG-3' 5'-AGAAGAGAGGCTGTGTGTTG-3'	58	429
24	5'-AACACACAGCCTCTCTTCTG-3' 5'-AGGAAACTGCGCTGTAACAG-3'	58	393
25	5'-GCAGTTTCCTCTGTCTGGTCAC-3' 5'-TCACAGCTTGGAGGGCTGGCTG-3'	58	442
26	5'-GGCCATCCCATTAATCCTTG-3' 5'-GATGCCCTGACAGAATCCTG-3'	58	370
27 & 28	5'-CCTGGGCATGCTTGAACTTG-3' 5'-CAGACCAAGTTCTGTCCAAG-3'	58	522
29	5'-CTTGGACAGAACTTGGTCTG-3' 5'-CATCCTGGCGGAAGCAAATG-3'	58	419
30	5'-AGCCCTCTCAGAGGGTTCTTG-3' 5'-ACAGCACCAGGAGCCCAGGCAG-3'	58	249
31	5'-CAAGCCTGTGACACTTTCAG-3' 5'-GATTGTGGGTTAGACTAGGG-3'	58	351
32	5'-CCTCACTTTGGTCTTACTGG-3' 5'-CGATTCAGAGCCCCGAAAAG-3'	58	440
33	5'-CTTTTCGGGGCTCTGAATCG-3' 5'-CTCTCTGCCAGGGAGAAATG-3'	58	391
34	5'-TTGATGGATAGGGTTCCATGGG-3' 5'-AGGGCCTAGAGGTATGCAAAG-3'	58	488
35	5'-GCTTGTTTAAGACCCTTCTGGG-3' 5'-TAACCACTGGTCTAGACTCCTG-3'	58	363
36	5'-TTAGCCACCCTGGAACACTG-3' 5'-GGAAGATTGAGGAAGAACGAGG-3'	58	352
37	5'-TCCCTGAGCTGCCTCCCCCTAC-3' 5'-AGGGTCCTCCTGACACCGTC-3'	58	267
38	5'-TACGTCCTCTTTCCCTCCTTTCTG-3' 5'-TGCCCTCATCTACAGCCAAG-3'	58	393
39	5'-CATCCTTCCACTCCTCCATC-3' 5'-AGCTTCACAGGTGGTCTCAG-3'	58	325
40 & 41	5'-GTGCCCCTGGGTGGAGGCTG-3' 5'-ACTGTGGCCAGGCCTGGTTTG-3'	58	410

Exons 12 & 13, 16 & 17, 27 & 28, and 40 & 41 were amplified as a single amplicon.

Sequence-derived electropherograms from the 16 individuals were compared with the published *CHD5 *sequence using Sequencher v4.6 software (Gene Codes Corp., Ann Arbor, MI).

### Computational analysis of single nucleotide polymorphisms

PMUT [20] online tool was used to assess the potential functional effects of missense changes found in *CHD5*. PMUT utilizes neural networks trained with a large database of neutral and disease-associated human mutations to predict the effects of single amino acid substitutions. The user inputs the reference amino acid sequence of the protein and designates the position of the amino acid substitution of interest. The output is a pathogenicity index ranging from 0 to 1 (indices > 0.5 predict a pathologic change and < 0.5 a neutral change) and a confidence index ranging from 0 (low) to 9 (high) [20].

ESEfinder 3.0 [21] was used to scan the genomic region containing SNPs found only among affected individuals in the screening panel for the identification of splice enhancer binding sites in the wildtype sequence. A comparison was then made with the variant sequence to determine if the single nucleotide substitution created a new splice enhancer binding site or destroyed an existing binding site.

## Results

**Figure 1:**
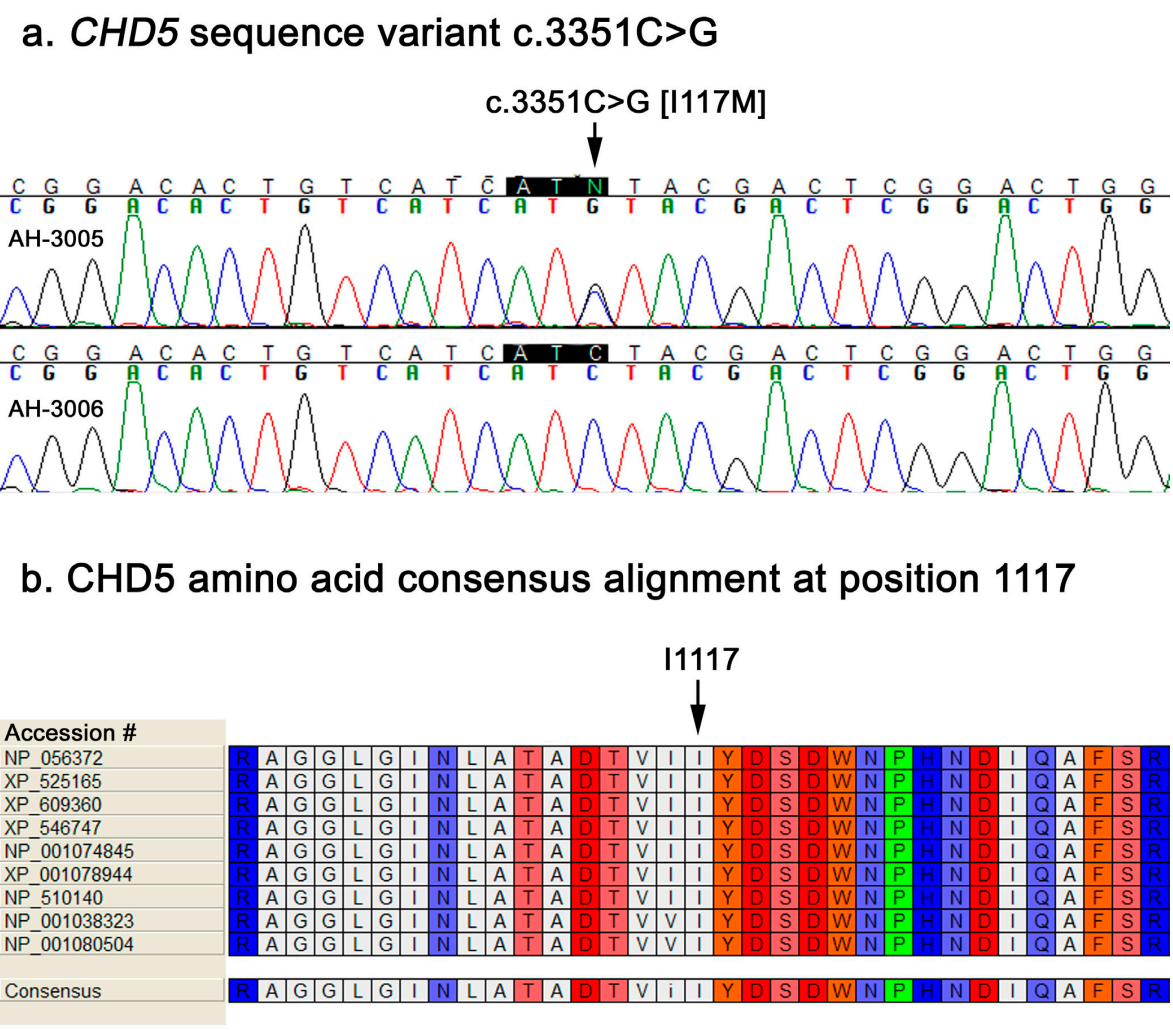
***CHD5 *sequence and amino acid alignment**. a. *CHD5 *exon 22 sequence variant c.3351C>G [I1117M] in affected individual AH-3005 compared to wildtype sequence in unaffected individual AH-3006. b. CHD5 amino acid consensus alignment at position 1117. Comparison of human CHD5 protein with mammalian orthologs and nonmammalian paralogs. NP_001074845, Chd5 Mus musculus; NP_001038323, Chd4 Danio rerio; NP_001080504, Chd4 Xenopus laevis, NP_056372, CHD5 Homo sapiens; NP_510140, Chd-3 Caenorhabditis elegans; XP_001078944, Chd5 Rattus norvegicus; XP_525165, Chd5 Pan troglodytes; XP_546747, Chd5 Canis lupus familiaris; XP_609360, Chd5 Bos Taurus.

All coding exons and flanking intronic sequence were examined. Six single nucleotide polymorphisms (SNPs) located in introns and four in the coding region (cSNPs) were detected in one or more affected individuals in the screening panel (Table 3). Among the 10 SNPs detected, four were previously identified in the dbSNP database and have an assigned rs identification number [22] and six are newly discovered (Table 3). Of the four cSNPs, three are synonymous and one is a nonsynonymous nucleotide change leading to a substitution of isoleucine for methionine at position 1117 (I1117M, Table 3). I1117M variant was detected in family AH in affected individual (AH-3005) and was not present in the unaffected individual (AH-3006) (Fig. 1a) or among the remainder of individuals in the screening panel (Table 1). Isoleucine 1117 is located in the C-terminal DNA binding domain of *CHD5*. Amino acid sequence alignment of the reference CHD5 protein (NP_056372) with mammalian Chd5 orthologs and non-mammalian paralogs (i.e. Chd-3 C. elegans, Chd4 in Danio and Xenopus) revealed high conservation of isoleucine at position 1117 (Fig. 1b). Computational analysis of the potential deleterious effects of the I1117M variant using the online tool PMUT predicted that this is a neutral substitution (index 0.049) with a reliability index of 9 (high). Scanning the genomic region of the four cSNPs (Table 3) with ESEfinder 3.0 [21] found a potential change in the c.531G>A (P177P) variant. The G to A nucleotide substitution at position 531 may potentially eliminate two SF2/ASF binding sites and create a new SC35 binding site in this region. There were no changes in exonic splice enhancer binding sites detected by ESEfinder 3.0 among the other three cSNPs.

**Table 3:** *CHD5 *sequence variants found among one or more affected in the screening panel

**Location**	**Nucleotide change**	**Amino acid change**	**db SNP ID**
Exon 5	c.531G>A	None [P177P]	Not in database
Intron 8	IVS8+41C>A	None	rs41279496
Intron 11	IVS11-7G>C	None	rs17489787
Intron 13	IVS13-17C>T	None	Not in database
Exon 15	c.2379C>T	None [N793N]	rs2273032
Exon 22	c.3336G>A	None [A1112A]	rs17029184
Exon 22	c.3351C>G	I1117M	Not in database
Intron 36	IVS36-38C>T	None	Not in database
Intron 36	IVS36-49C>T	None	Not in database
Intron 39	IVS39+34C>T	None	Not in database

dbSNP, single nucleotide polymorphism database.

Among the six intronic SNPs associated with affected individuals in the screening panel, two located in introns 8 and 11 have been previously identified (rs41279496, rs17489787) in dbSNP [22] and four are newly discovered in this study (Table 3). Computational search of the genomic region containing these six intronic SNPs for exonic splice enhancer sequence with the online tool ESEfinder 3.0 [21] found no destruction of existing splice enhancer binding sites or creation of additional binding sites by these SNPs.

Sequencing of additional affected and unaffected members from family AH revealed that the I1117M variant is present in the unaffected mother of AH-3005, but not in his affected father nor in the four remaining CMM/DN affected individuals in this family. Sequence analysis of the remainder of the intronic SNPs and synonymous cSNPs detected in the screening panel (Table 3) among additional affected and unaffected family members from each respective family revealed that these polymorphisms did not co-segregate with the CMM/DN trait.

## Discussion

The search for additional major susceptibility genes for familial melanoma has remained elusive. Currently two genes (*CDKN2A*, *CDK4*) have been identified and together account for ≤ 40% of all CMM families. Thus, a majority of CMM families do not have a defined CMM susceptibility gene. Linkage analysis of *CDKN2A, CDK4 *mutation negative families identified a susceptibility locus at 1p22 [12], however, no candidate gene has been identified. Prior linkage studies identified a subset of CMM/DN families with simultaneous linkage to 9p21 and 1p36 [15]. Loss of heterozygosity at 1p36 was originally found in neuroblastoma [23] and has subsequently been reported in many types of human tumors [24] suggesting the presence of a tumor suppressor gene(s) in this region. Somatic deletion of the 1p36 locus occurs in a wide range of solid and lymphoid tumors [24] and has been observed in nodular, metastatic and superficial spreading melanomas [16,17]. Therefore the loss of a common tumor suppressor gene or a combination of several genes in this region may predispose to tumor development or contribute to tumor progression [24,25].

Recently, *CHD5 *was identified as a candidate tumor suppressor gene [18,24] by the use of Cre-loxP site-specific recombinant technology to generate a region of gain or loss of mouse chromosome 4 corresponding to the human 1p36 locus. Bagchi *et al *[18,24] was able to demonstrate that mouse cells with an extra copy of chromosome 4 corresponding to human 1p36 exhibited enhance cellular senescence. The observed cellular senescence was rescued by RNAi-mediated knockdown of p53. Mouse cells deficient in *Chd5 *due to loss of one copy by Cre-loxP-mediated recombination expressed decreased levels of p53, p16 and p19. Subsequent experiments showed that cellular proliferation can be restored by depletion of p19 suggesting that *Chd5 *regulated p53 expression and that cell growth was directed through chromatin remodeling and control of gene expression at the *CDKN2A *(p16/p19) locus.

Among the eight CMM/DN families screened for *CHD5 *mutations in this study, only one family (AH) was found to have a missense coding change. However, upon in depth mutation analysis of family AH with additional affected and unaffected family members, the I1117M variant was found to be inherited from the unaffected mother. Family AH consists of six CMM/DN affected individuals. The CMM/DN phenotype is inherited through the paternal side of the family. Neither the affected father nor additional affected individuals carried the I1117M variant. This finding strongly suggests that the I1117M variant is not associated with the CMM/DN phenotype. Sequence analysis of the remaining intronic SNPs and synonymous cSNPs found in the screening panel (Table 3) among additional affected and unaffected individuals from their respective CMM/DN kindreds revealed that none of these variants showed complete co-segregation with the CMM/DN phenotype. Taken together, these results did not support *CHD5 *as a melanoma susceptibility gene in these eight families.

Limitations of this study include small sample size. Also we did not possess a source of mRNA to study *CHD5 *expression in tumor or compare melanocyte expression of *CHD5 *between affected individuals and controls. To our knowledge, the eight families screened represent all the known CMM families linked to 1p36. We limited our *CHD5 *mutation screen to the coding regions and flanking splice sites, thus changes to promoter, enhancer and micro-RNA binding sites may have been missed. We caution that our findings pertain only to the eight families screened in this study and should not be generalized to other CMM families.

## Conclusion

In conclusion, we have not found evidence that *CHD5 *is a major melanoma susceptibility gene among the eight CMM/DN families screened. We are not aware of additional CMM families linked to the 1p36 locus. Thus, mutation screening of more 1p36-linked families may be challenging. Given the small number of families analyzed, a rare mutation in *CHD5 *may have been missed.

## Abbreviations

*CDKN2A*: cyclin-dependent kinase inhibitor 2A; *CDK4*: cyclin-dependent kinase 4; *CHD4*: chromodomain helicase DNA-binding protein 4; *CHD5*: chromodomain helicase DNA-binding protein 5; CMM/DN: cutaneous malignant melanoma and dysplastic nevi; cSNP: coding SNP; dbDNP: single nucleotide polymorphism database; *MC1R*: melanocortin 1 receptor; p16: alternate designation for *CDKN2A*; p19/ARF: designation for alternate-spliced exon 1β transcript of *CDKN2A*: SNP, single nucleotide polymorphism.

## Competing interests

The authors declare that they have no competing interests.

## Authors' contributions

DN provided the bioinformatics support, designed and carried out the sequencing of *CHD5*. He collated, analyzed the sequencing data and drafted the manuscript. AG generated the hypothesis for this study, reviewed the sequence variants and affection status among the eight CMM/DN pedigrees and derived the segregation analysis of the *CHD*5 variants. XY and AG identified the eight CMM/DN families showing linkage to 1p36. MT identified and evaluated the CMM/DN families. All authors read, contributed to and approved the final manuscript.
